# Infectious disease surveillance system in Pakistan: challenges and way forward

**DOI:** 10.1186/s41182-022-00439-y

**Published:** 2022-07-19

**Authors:** Taha Gul Shaikh, Summaiyya Waseem, Syed Hassan Ahmed, Sarya Swed, Mohammad Mehedi Hasan

**Affiliations:** 1 Dow University of Health Sciences, Karachi, Pakistan; 2 Faculty of Medicine, Aleppo University, Aleppo, Syria; 3 Department of Biochemistry and Molecular Biology, Faculty of Life Science, Mawlana Bhashani Science and Technology University, Tangail, 1902 Bangladesh

**Keywords:** Infectious disease, Surveillance, Nationwide approach, Pakistan, Public health

## Abstract

Infectious Disease Surveillance (IDS) in the community is essential to prevent, control, and detect outbreaks. A strong surveillance system is a need of time for low to middle-income countries like Pakistan where around half of the reported deaths are due to infections that can be easily prevented in the presence of a surveillance system. Although Pakistan has IDS which monitors and collects the data on several infectious diseases, the system is unreliable, inadequate, and substandard. As a result, dengue, tuberculosis, malaria, hepatitis B and C, and many other infectious diseases are still prevalent in Pakistan and unfortunately, the numbers are still rising. In this commentary, we have tried to highlight the problems the country is facing to establish a proper and self-sustainable surveillance system and have recommended some steps the relevant stakeholders should consider taking to make healthcare better in Pakistan.

Dear Editor,

Infectious disease surveillance, a prime epidemiological tool, involves the collection, analysis, and interpretation of an enormous volume of data to identify disease patterns, outbreaks, and new pathogens along with the monitoring and elimination of diseases [1]. The National Institute of Health (NIH), formerly known as the National Health Center, was given an autonomic status in 1980. Since then, it is involved in multidisciplinary public health activities including diagnostic services, research, etiology, and epidemiology of infectious diseases in Pakistan. In 1998, an epidemic investigation cell, later renamed as Field Epidemiology and Disease Surveillance Division, was set up under the NIH to counter outbreaks and epidemics, take part in national and international public health events, and provide necessary data to the concerned stakeholders [2]. Despite its existence for more than four decades, there is still a high prevalence of infectious diseases in Pakistan. According to World Health Organization (WHO), the country is facing the endemicity of hepatitis B and C, and is amongst the top five countries with tuberculosis, and has an HIV prevalence of 21% [3]. Hence, preventable diseases such as tuberculosis, meningitis, malaria, hepatitis B and C, and typhoid account for nearly half of the deaths in the country [4].

Efforts have been made on both the national and provincial levels to combat this issue. A Field Epidemiology and Laboratory Training program, launched in 2006, contributed to the formation of a surveillance system for acute viral hepatitis and started a campaign to eradicate polio in the country in 2009 and 2011, respectively. Similarly, in 2015, the program established six disease surveillance and response units throughout the country [5]. More recently, a 3-month training course was started to train public health care workers who are involved in surveillance work [6].

In July 2013, the Disease Surveillance System was implemented in the largest province of the country (Punjab), aiming to monitor and prevent future epidemics. The data, collected from all the healthcare facilities, are reviewed by a committee that then issues weekly bulletins and alerts. The system helped authorities prevent an outbreak of measles in 2012 and H1N1 influenza in 2015. Currently, the committee focuses on 26 communicable diseases listed by the WHO; however, the system is being upgraded to monitor 81 diseases [7]. Although the steps taken by the provincial government of Punjab are commendable, efforts made by other provinces are inadequate and substandard.

Numerous factors are hindering the development of an integrated disease surveillance system in Pakistan. The major issue in building an effective surveillance system is limitation of financial and logistic resources. The country's environment and terrain render it difficult to provide surveillance tools, communication devices, vehicles, and office supplies, in all the districts, especially in remote areas. The financial constraints can be judged by the fact that in 2019, Pakistan allotted only 1% of the GDP to the health sector, contrary to the WHO-adopted resolution of allocating a minimum of 5% in developing countries [8]. Other reasons include inconsequential importance given to such systems primarily due to non-recognition, lack of budget allotment, and workload. Similarly, limited trained staff who know the importance of such systems in the healthcare facility and the lack of coordination between the provincial and federal government to develop an integrated disease surveillance system and response (IDSR) is compromising the ability of the country’s healthcare system to become self-resilient and sustainable [4].

The fate of the country’s healthcare system and the establishment of national infectious disease surveillance cannot be achieved overnight. A highly competent team, robust logistic and financial support, and in-depth research are required to identify the already existing loopholes, and to devise and implement a new strategic plan. The country is in dire need of an effective active and passive surveillance system. Requiring significant financial and human resources, active surveillance is expensive, however, it is the most accurate. The passive surveillance system requires medical professionals and healthcare facilities to report the cases, but is less reliable owing to the chances of missing the cases due to reporting structure [9]. It would be better for resources limited countries like Pakistan to focus more on passive surveillance and gradually shift towards the active system along with proper induction of epidemiologists to fill positions all over the country by offering better job benefits and salaries to achieve the best out of the already existing tools. There is also a strong need to develop an independent regulatory body that should be responsible for integrating both private and public sector hospitals into an effective surveillance system.

Pakistan can also take advantage of the pre-existing syndromic surveillance systems and replicate them in each district. In 2005, WHO along with the Ministry of Health Pakistan established the Disease Early Warning System in earthquake-affected areas. Later, this system was used to cover flood-affected areas and conflict-affected areas in northern Pakistan in 2005 and 2009, respectively [10].

The economic woes to develop a competent IDSR can be achieved by collaborating with international partners. Pakistan should collaborate with the Centers for Disease Control and Prevention and other potential foreign partners for technical, financial, and training support. Laboratory capacity along with quality assurance programs should also be increased on an urgent basis. Proper data communication methods and a change in the attitude of healthcare personnel are also need of time.

## Data Availability

Not applicable.

## Abbreviations

NIH: National Institute of Health
HIV: Human immunodeficiency virus
WHO: World Health Organization
IDSR: Integrated disease surveillance system and response
GDP: Gross domestic product
IDS: Infectious Disease Surveillance
